# Association of *TP53* Codon* 72 Arg>Pro* Polymorphism with Breast and Lung Cancer Risk in the South Asian Population: A Meta-Analysis

**DOI:** 10.31557/APJCP.2020.21.6.1511

**Published:** 2020-06

**Authors:** Sarah Jafrin, Md Abdul Aziz, Shamima Nasrin Anonna, Tahmina Akter, Nura Ershad Naznin, Sharif Reza, Mohammad Safiqul Islam

**Affiliations:** *Department of Pharmacy, Faculty of Science, Noakhali Science and Technology University, Noakhali, Bangladesh. *

**Keywords:** Arg72Pro, breast cancer, lung cancer, meta-analysis, TP53 polymorphism

## Abstract

**Background:**

A transversion missense polymorphism of the* TP53* tumor suppressor gene at the codon 72 codes proline instead of arginine causes an altered p53 protein expression and has been found to be associated with an elevated risk of various cancer; especially breast and lung cancer. As the previous case-control studies on the South Asian population have shown controversial results, we performed a meta-analysis to evaluate a precise estimation of the relationship between the *TP53 Arg72Pro *polymorphism with breast and lung cancer.

**Methods:**

A total of 12 related studies on the South Asian population have been included through comprehensive database searching. Six studies were selected for breast cancer meta-analysis involving 950 cases and 882 controls; the other six studies were for lung cancer meta-analysis including 975 cases and 1397 controls. The results have been determined by using the Review Manager (RevMan) 5.3. Additionally, the stability of our analysis was assessed by heterogeneity, publication bias analysis and sensitivity testing.

**Results:**

A significantly increased risk of breast cancer was found in Pro allele (Pro vs. Arg), co-dominant model 2 (Pro/Pro vs. Arg/Arg), dominant model (Pro/Pro + Arg/Pro vs. Arg/Arg). In case of lung cancer, significantly increased risk was found in the allele, co-dominant 1, co-dominant 2, co-dominant 3, dominant, and recessive models. No association with other genetic models with breast and lung cancer risk was found in the South Asian population.

**Conclusions:**

Our results indicate that *TP53 Arg72Pro* polymorphism is a risk factor for the development of breast cancer and lung cancer in the South Asian population.

## Introduction

Cancer is one of the most severe human diseases with a high rate of morbidity results from uncontrolled, abnormal cellular proliferation due to genetic mutation. The global cancer rate is rapidly rising and in 2018, new cancer cases rose to 18.1 million, with 9.6 million deaths. Lung cancer and female breast cancer are the leading types of cancers worldwide in terms of the number of new cases and deaths. In 2018, approximately 2.1 million diagnoses were estimated for both types of cancers, contributing about 11.6% of the total cancer incidence burden (Ferlay et al., 2018; Meek, 2015). According to Globocan 2018, among the total number of cancer cases, 13.6% was breast cancer (ranked 1) and 8% was lung cancer (ranked 2) cases in the WHO Southeast Asia region (Ferlay et al., 2018).

Positive regulation of tumor suppressor genes inhibits the formation of a tumor by halting various mechanisms during the cell cycle. *TP53* is one of the major tumor suppressor genes that provides a critical barrier to the development of cancer by blocking cellular proliferation or eliminating cancer cells. Mutation of the *TP53* gene interferes with its tumor suppression function and has a critical role in the formation of human cancer, whereas a mutant *TP53* gene is relatively higher in cancer patients than in the control population (Mantovani et al., 2019; Sreeja et al., 2008). The majority of *TP53* mutations are transversion missense mutations that result in the formation of different amino acids rather than the normal one due to purine and pyrimidine nucleotide substitution. The position of mutation lies within the part of the gene that encodes the site-specific DNA-binding domain that causes false transcription and failure in tumor suppression. Mutant p53 proteins are generally very stable in cancer cells than the normal p53 protein and can be detected easily by immunohistochemistry (Rivlin et al., 2011; Sreeja et al., 2008; Khan et al., 2015)

About 30% of *TP53* missense mutations are found during cancer development in the highly mutable region of the human gene – *CpG* region, where the frequencies of cysteine and guanine base are comparatively higher. The region contains essential transcription factor binding site that in turn controls structural and chemical contact with the combined effect of p53 protein and specific DNA sequences that develop specific p53 response elements. The most extensively studied SNP *(rs1042522*) is a transversion polymorphism of G nucleotide to C nucleotide in codon 72 in exon 4 at the second position, leading to the formation of proline rather than arginine (Pro72 protein variants). The gene product of Arg72 variant is more efficient in apoptotic activity than the mutant Pro72 variant of *TP53* gene. The allelic frequencies of *TP53* codon 72 differ among different ethnic populations of the world that changes cancer susceptibility in these populations (Petitjean et al., 2007).

Numerous studies have conducted research over the association of *TP53* codon* 72 rs1042522* polymorphism with breast and lung cancer risk, but results have been contradictory and no clear evidence has been found. The conflicting results may arise from insufficient data, small sample size or different study designs. A single study is less potential to make a reliable outcome due to these limitations (Petitjean et al., 2007; Huang et al., 2003).

Moreover, several meta-analyses have been conducted on *TP53* codon 72 polymorphism and its relation to breast cancer or lung cancer, mostly on the Caucasian and Asian population, including Japanese (Huang et al., 2003), Chinese (Liu et al., 2013; Chua et al., 2010) and Taiwanese (Wang et al., 1999) regions. But no previous meta-analysis has been conducted on the South Asian population, including Bangladeshi, Indian, Pakistani population, although the prevalence of lung cancer and breast cancer is extremely high in South Asia (Ferlay et al., 2018). A high degree of variation in the genetic profile occurs due to geographic and ethnic variation that changes the prevalence of the disease in different regions of the world (Mantovani et al., 2019; Chua et al., 2010; Wang et al., 1999). Even in the same ethnicity, two different studies may show the opposite results (Sreeja et al., 2008; Jain et al., 2005). The ancestry of the Indian subcontinent population is unique and admixture of the population was observed from other parts of the world (Islam et al., 2013; Mehra et al., 2010).

For this reason, it was necessary to find out the relation between lung cancer and breast cancer with *TP53* codon 72 polymorphism in the South Asian population. To evaluate the potential correlation comprehensively between the *TP53 Pro72Arg* polymorphism with the lung and breast cancer risk in South Asian population, and to improve our understanding for a more precise characterization of the *Pro72Arg* polymorphism, we designed a systematic review and meta-analysis of 12 candidate studies conducted on the South Asian population.

## Materials and Methods

*Methodology*

*Literature search for identification of relevant studies*

**Figure 1:**
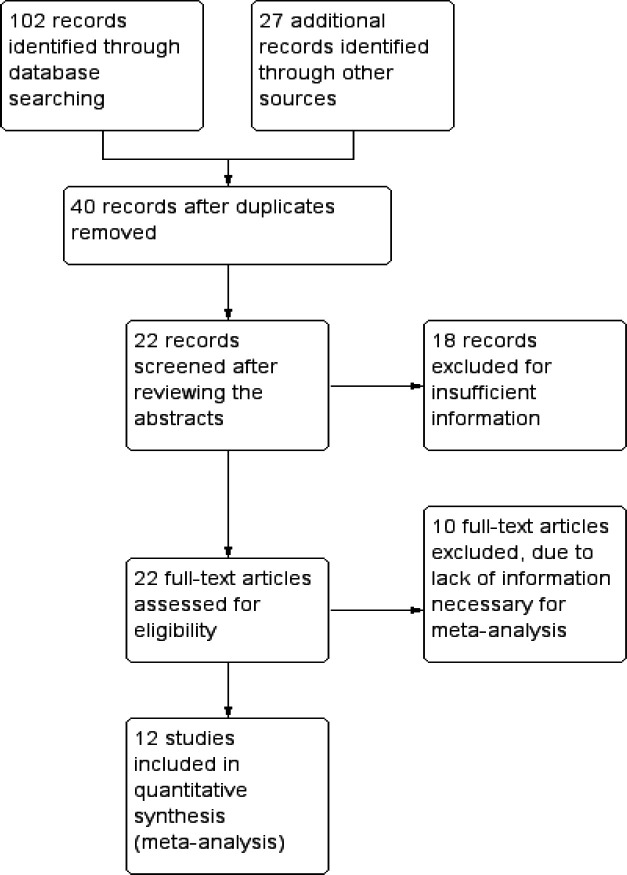
Flow Chart of Literature Screening and Selection in the Meta-Analysis

A systemic comprehensive search was performed in Pubmed, Web of Science, Scopus and Google scholar. Two independent researchers searched the publications up to September 2019 using either single or combination of given following keywords- “Arg72Pro polymorphism”, “p53 codon 72 rs1042522 polymorphism”, “p53 and lung cancer or breast cancer”. The summary of the process of selecting eligible studies was presented in Figure 1. The inclusion criteria of the eligible studies were: 1) studies must be published in English, 2) original case-control studies correlating *TP53* codon* 72 Arg>Pro* polymorphism with lung or breast cancer conducting on the South Asian population 3) studies providing information regarding genotype and allelic frequencies of both cases and controls, odds ratio with 95% confidence interval (CI) values. The exclusion criteria were: 1) studies published in an animal model or cell line 2) studies having only the data of cases 3) lacking any genotype frequency for *TP53* codon *72 Arg>Pro* polymorphism 4) studies conducted on the other ethnic groups except the South Asian population.

*Data extraction*

Two researchers (SF and MSI) independently collected the first author’s name, year of publication, sample size, the ethnicity of the cases and controls, genotype and allele frequencies for each case and control.

*Statistical analysis*

RevMan (Version 5.3) software (http://www.cochrane.org/revman) was used to conduct this meta-analysis. All of the data in the studies are dichotomous data. The outcome has been expressed as odds ratios (ORs) with 95% confidence intervals (CIs) to assess the association between the polymorphisms and cancer. OR>1 indicates increased risk and <1 indicates reduced risk.

Chi-square tests of control group data was performed for each study to determine the Hardy-Weinberg equilibrium (HWE). The HWE p values are shown in Table 1. Three studies related to lung cancer deviated from HWE as p<0.05 (Table 1). ORs and CIs were pooled to evaluate the association between the *TP53* polymorphisms and the risk of breast or lung cancer. For each type of cancer to find the association with polymorphism, the ORs were calculated for co-dominant, dominant, recessive, overdominant, and allele genetic models. Heterogeneity in the forest plot also assessed using the I2 statistic and the Cochrane chi-square Q-test. The significance level of heterogeneity is p<0.10 and I2>50%. The fixed-effects model (the Mantel-Haenszel method) was applied for meta-analysis when heterogeneity was not significant. The random-effects model was used to estimate pooled ORs when heterogeneity values were significant. Z-test was performed to evaluate the significance of the pooled OR and p<0.05 was considered to be statistically significant (Hashemi et al., 2018). Publication bias was appraised using Begg’s funnel plot. The degree of asymmetry of Begg’s funnel plot was measured using Egger’s linear regression test and the level of significance of publication bias was p<0.05. The sensitivity analysis was done to examine the influence of each study on the pooled results by removing studies one by one. The significance level of publication bias was p<0.05. 

## Results

*Study characteristics*

A total of 12 case-control studies were included in this meta-analysis to find out the association of *TP53* codon 72 *Arg/Pro* polymorphism with lung cancer and breast cancer in the South Asian population based on the inclusion criteria (Shabnaz et al., 2016; Hashemi et al., 2018; Aziz et al., 2013; Suresh et al., 2011; Syeed et al., 2010; Ihsan et al., 2011; Tilak et al., 2013; Chowdhury et al., 2015; Mostaid et al., 2014; Saikia et al., 2014; Sharma et al., 2014). Among these, six studies evaluated the association for breast cancer (950 cases and 882 controls) and the other six studies for lung cancer (975 cases and 1,397 controls). All the studies were performed in the various ethnic groups of South Asia. Table 1 explains the characteristics, genotype frequency, allelic frequency and HWE p-value (for control only) of the selected studies.

*Association of TP53 codon 72 Arg>Pro polymorphism with breast and lung cance*r

**Figure 2:**
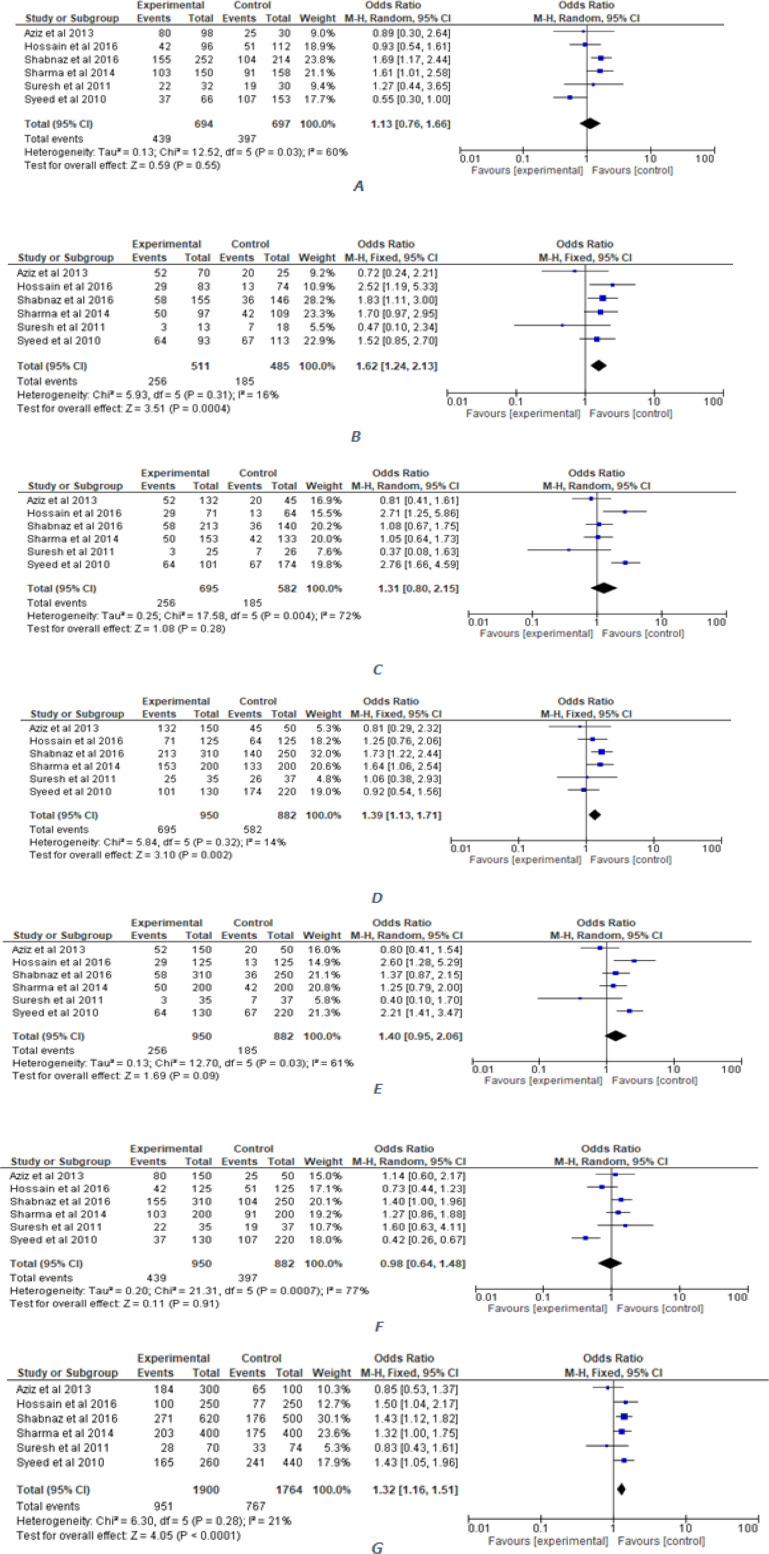
Forest Plot Showing the Association between TP53 Codon 72 Polymorphism and Breast Cancer in the Study. population under following models; A) Arg/Pro vs Arg/Arg; B) Pro/Pro vs Arg/Arg; C) Pro/Pro vs Arg/Pro; D) Pro/Pro + Arg/Pro vs Arg/Arg; E) Pro/Pro vs Arg/Pro +Arg/Arg; F) Arg/Pro vs Arg/Arg + Pro/Pro & G) Pro vs Arg.

**Figure 3.**
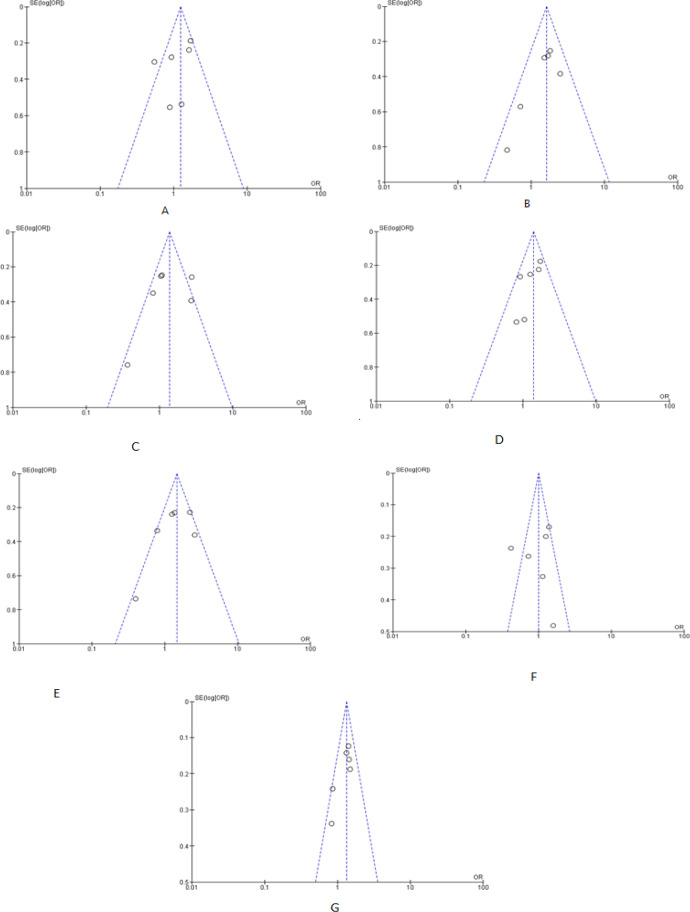
Funnel Plots for the Association between TP53 Codon 72 Polymorphism and Breast Cancer in the Study Population under Following Models; A) Arg/Pro vs Arg/Arg; B) Pro/Pro vs Arg/Arg; C) Pro/Pro vs Arg/Pro; D) Pro/Pro + Arg/Pro vs Arg/Arg; E) Pro/Pro vs Arg/Pro + Arg/Arg; F) Arg/Pro vs Arg/Arg + Pro/Pro & G) Pro vs Arg

**Figure 4:**
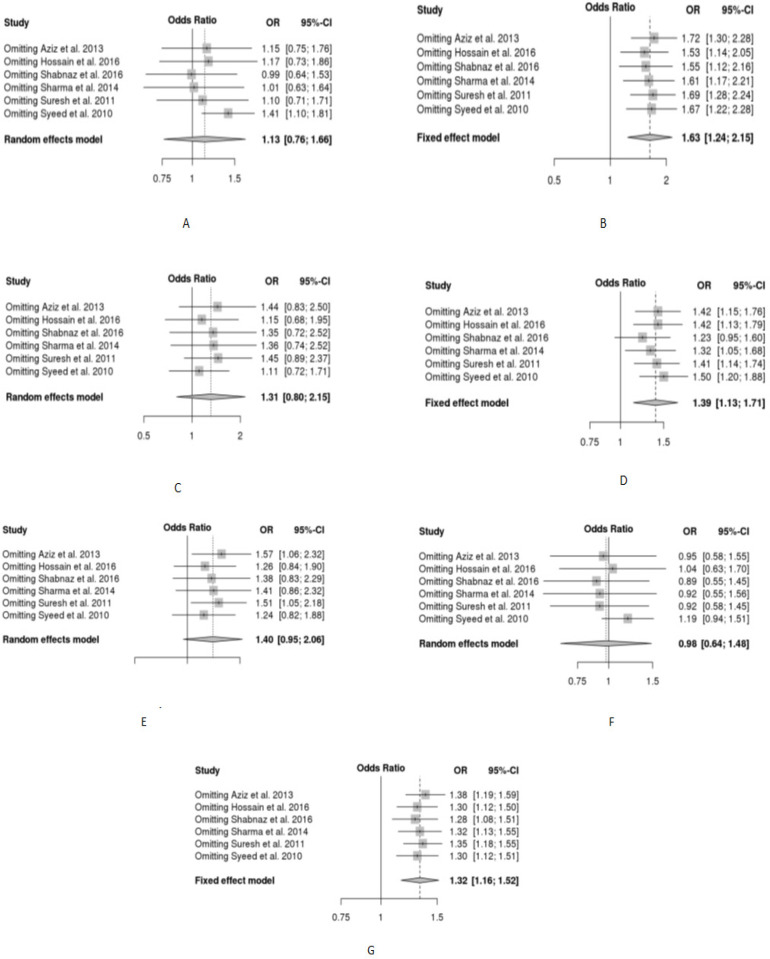
Sensitivity Analysis for the Studies on TP53 Codon 72 Polymorphism and Breast Cancer Using Different Genetic Models; A) Arg/Pro vs Arg/Arg; B) Pro/Pro vs Arg/Arg; C) Pro/Pro vs Arg/Pro; D) Pro/Pro + Arg/Pro vs Arg/Arg; E) Pro/Pro vs Arg/Pro + Arg/Arg; F) Arg/Pro vs Arg/Arg + Pro/Pro & G) Pro vs Arg

The association between breast and lung cancer with *72 Arg>Pro *polymorphism was mentioned in Table 2. The Forest plots, Funnel plots and plot for sensitivity analysis for breast cancer were shown in Figure 2, Figure 3 and Figure 4 respectively, whereas these plots were shown in Figure S1, Figure S2 and Figure S3, respectively for lung cancer.

*Association of TP53 codon 72 Arg>Pro polymorphism with breast cancer*

To measure the association of breast cancer with *TP53 *codon 72 polymorphism, we pooled together six studies related to the breast cancer (950 cases and 882 controls). As shown in Table 2 and Figure 2, these analyses suggest a significant risk of breast cancer in various genetic models. A significant increased risk was found in allele (Pro vs. Arg; OR=1.32, 95%CI=1.16-1.52, p=5.26x10^-5^), dominant (Pro/Pro + Arg/Pro vs. Arg/Arg; OR=1.39, 95%CI=1.13-1.71, p=1.96x10^-3^), and co-dominant 2 (Pro/Pro vs. Arg/Arg; OR=1.63, 95%CI=1.24-2.15, p=4.76x10^-4^) genetic models. Though co-dominant models 1 and 3 (Arg/Pro vs. Arg/Arg; OR=1.13, 95%CI=0.76-1.66, p=0.553; Pro/Pro vs. Arg/Pro; OR=1.31, 95%CI=0.81-2.15, p=0.282) and recessive model (Pro/Pro vs. Arg/Pro + Arg/Arg; OR=1.40, 95%CI=0.95-2.06, p=0.091) showed increased risk, these models were not statistically significant to develop breast cancer. Overdominant model (Arg/Pro vs. Arg/Arg + Pro/Pro; OR=0.98, 95%CI=0.64-1.49, p=0.910), on the contrary, showed decreased susceptibility for the breast cancer that was not statistically significant. 

*Association of TP53 codon 72 Arg>Pro polymorphism with lung cancer*

Through the meta-analysis of six studies related to lung cancer including 975 cases and 1397 controls, it was found that (Table 2 and Figure S1) Pro allele model (Pro vs. Arg; OR=1.57, 95%CI=1.21-2.05, p=6.91x10^-4^), co-dominant model 1 (Arg/Pro vs. Arg/Arg; OR=1.38, 95%CI=1.14-1.68, p=9.26x10^-4^), co-dominant model 2 (Pro/Pro vs Arg/Arg; OR=2.28, 95%CI=1.47-3.55, p=2.50x10^-4^), co-dominant model 3 (Pro/Pro vs .Arg/Pro; OR=1.48, 95%CI=1.18-1.86, p=6.94x10^-4^ ), dominant model (Pro/Pro + Arg/Pro vs Arg/Arg; OR=1.70, 95%CI=1.24-2.34, p=1.04x10^-3^) and the recessive (Pro/Pro vs. Arg/Pro + Arg/Arg; OR=1.81, 95%CI=1.28-2.56, p=7.82x10^-4^) of *TP53* codon 72 polymorphism were significantly associated with lung cancer development. No statistically significant association was found with overdominant model (Arg/Pro vs Arg/Arg + Pro/Pro; OR=1.06, 95%CI=0.90-1.26, p=0.477).

*Heterogeneity and Publication bias*

Heterogeneity was checked and the co-dominant model 1 (Arg/Pro vs. Arg/Arg) co-dominant model 3 (Pro/Pro vs. Arg/Pro), overdominant model (Arg/Pro vs. Arg/Arg + Pro/Pro) and recessive model (Pro/Pro vs. Arg/Pro + Arg/Arg) showed heterogeneity (I^2^> 50) in case of breast cancer. Whereas co-dominant model 2 (Pro/Pro vs. Arg/Arg), dominant model (Pro/Pro + Arg/Pro vs. Arg/Arg), recessive model (Pro/Pro vs. Arg/Pro + Arg/Arg) and allele model (Pro vs. Arg), showed heterogeneity in case of lung cancer (Table 2).

Begg’s funnel plots were generated to evaluate the risk of publication bias for each genetic model as visual guidance (Figure 3 and Figure S2). No asymmetry was visualized in funnel plot analysis that indicates the absence of publication bias among the studies. Moreover, Egger’s linear regression analysis also illustrated that there is no publication bias exists in any genotype and allele models as all the p-value of bias test >0.05 (Table 2).

*Sensitivity analysis*

The reliability and stability of the performed meta-analysis were ensured by performing a sensitivity test that identifies whether the pooled OR was influenced by each included study. Sensitivity analysis was performed by the omission of the studies one by one (Figure 4 and Figure S3). The results ensured that the performed meta-analysis was stable and reliable.

**Table 1:** Genotypic and Characteristic Information of the Selected Studies for Meta-Analysis

Author	Country	Ethnicity	Cancer type	Genotyping method	Case/Control	Case					Control					
						Arg/Arg	Arg/Pro	Pro/Pro	Arg	Pro	Arg/Arg	Arg/Pro	Pro/Pro	Arg	Pro	HWE *P*-value
Aziz et al 2013	Pakistan	South Asia	Breast	PCR-RFLP	150/50	18	80	52	116	184	5	25	20	35	65	0.484
Hossain et al 2016	Bangladesh	South Asia	Breast	PCR-RFLP	125/125	54	42	29	150	100	61	51	13	173	77	0.632
Shabnaz et al 2016	Bangladesh	South Asia	Breast	PCR-RFLP	310/250	97	155	58	349	271	110	104	36	324	176	0.164
Sharma et al 2014	North India	South Asia	Breast	PCR-RFLP	200/200	47	103	50	197	203	67	91	42	225	175	0.285
Suresh et al 2011	North India	South Asia	Breast	PCR-RFLP	35/37	10	22	3	42	28	11	19	7	41	33	0.812
Syeed et al 2010	Kashmir	South Asia	Breast	PCR-RFLP	130/220	29	37	64	95	165	46	107	67	199	241	0.786
Sreeja et al 2008	India	South Asia	Lung	PCR-RFLP	211/211	70	84	57	224	198	98	76	37	272	150	0.002
Ihsan et al 2011	India	South Asia	Lung	PCR-RFLP	161/274	38	86	37	162	160	64	141	69	269	279	0.625
Tilak et al 2013	India	South Asia	Lung	PCR-RFLP	175/202	36	98	41	170	180	67	111	24	245	159	0.032
Saikia et al 2014	India	South Asia	Lung	PCR-RFLP	272/544	95	125	52	315	229	225	260	59	710	378	0.208
Mostaid et al 2014	Bangladesh	South Asia	Lung	PCR-RFLP	106/116	27	40	39	94	118	62	35	19	159	73	0.001
Chowdhury et al 2015	Bangladesh	South Asia	Lung	PCR-RFLP	50/50	12	19	19	43	57	21	18	11	60	40	0.077

**Table 2:** Meta-Analysis of the Association between TP53 codon 72 Arg>Pro Polymorphisms and Breast and Lung Cancer Risk in South Asian Population

Genetic Model	Test of Association			Test of Heterogeneity			Publication Bias
	OR	95% Cl	p	Model	Test of heterogeneity p-value	I^2^ (%)	p-val (Egger's test)
Breast cancer							
Arg/Pro vs. Arg/Arg	1.13	0.76-1.66	0.553	Random	0.028	60.08	0.323
Pro/Pro vs. Arg/Arg	1.63	1.24-2.15	4.76x10^-4^	Fixed	0.313	15.66	0.095
Pro/Pro vs. Arg/Pro	1.31	0.81-2.15	0.282	Random	0.003	71.55	0.585
Dominant model (Pro/Pro + Arg/Pro vs. Arg/Arg )	1.39	1.13-1.71	1.96x10^-3^	Fixed	0.321	14.42	0.103
Overdominant Model (Arg/Pro vs. Arg/Arg + Pro/Pro)	0.98	0.64-1.49	0.91	Random	0.0007	76.53	0.789
Recessive model (Pro/Pro vs. Arg/Pro + Arg/Arg)	1.4	0.95-2.06	0.091	Random	0.026	60.62	0.354
Allele contrast (Pro vs Arg)	1.32	1.16-1.52	5.26x10^-5^	Fixed	0.278	20.63	0.06
Lung cancer							
Arg/Pro vs. Arg/Arg	1.38	1.14-1.68	9.26x10^-4^	Fixed	0.168	35.86	0.165
Pro/Pro vs. Arg/Arg	2.28	1.47-3.55	2.50 x10^-4^	Random	0.008	67.84	0.438
Pro/Pro vs. Arg/Pro	1.48	1.18-1.86	6.94 x10^-4^	Fixed	0.246	25.1	0.718
Dominant model (Pro/Pro + Arg/Pro vs. Arg/Arg )	1.7	1.24-2.34	1.04x x10^-3^	Random	0.017	63.88	0.254
Overdominant Model (Arg/Pro vs. Arg/Arg + Pro/Pro)	1.06	0.90-1.26	0.477	Fixed	0.844	0	0.171
Recessive model (Pro/Pro vs. Arg/Pro + Arg/Arg)	1.81	1.28-2.56	7.82 x10^-4^	Random	0.028	60.12	0.427
Allele contrast (Pro vs Arg)	1.57	1.21-2.05	6.91x10^-4^	Random	0.0006	77.12	0.262

## Discussion

The *TP53 *gene encodes tumor suppressor protein that contains several domains essential for mediating its various functions. Among various domains, two important transactivation domains are TAD1 and TAD2, both located at the N-terminus of the protein. Due to polymorphism at codon 72 of *TP53*, Arginine gets replaced by Proline and *TAD2* overlaps with a Proline-rich domain that makes important contributions to repression, apoptosis and the response to γ –irradiation (Muller et al., 2014). Excess presence of Proline will change the overlapping tendency of *TAD2* as well as tumor suppression activity. During cancer development, mutation of the *TP53* gene not only results in the ablation of wild type *p53* tumor-suppressor activity but also gives rise to a mutant protein that encompasses oncogenic functions (Oren et al., 2010; Hou et al., 2013). *TP53 Arg72Pro* (*rs1042522* ) polymorphism occurs at exon 4 among the 11 exons of codon 72 of the *p53* tumor suppressor gene located on chromosome 17p13. The polymorphism causes the transition of CGC to CCC, leading to a differently functioning protein with a proline-rich domain at codon 72 (Arg72Pro). Studies have reported that the codon 72 polymorphism is associated with a risk for the development of cancer (Damin et al., 2006).

Many previous studies suggested that there may be a significant risk association of *TP53* codon 72 polymorphism with lung and breast cancer, yet the outcome was conflicting. A number of studies have reported that there is a significant association between *TP53* codon 72 polymorphism and breast cancer risk (Sjalander et al., 1996; Kalemi et al., 2005; Akkiprik et al., 2009; Buyru et al., 2003; Goncalves et al., 2014; Henriquez-Hernandez et al., 2009; Katiyar et al., 2003; Li et al.’ 2002; Baynes et al., 2007) and other studies showed that there is no significant association (Mabrouk et al., 2003; Khadang et al., 2007; Schmidt et al., 2007; Tommiska et al., 2005; Murata et al., 1996). To overcome this conflicting situation, we performed a meta-analysis to evaluate the association between *TP53 Arg72Pro* polymorphism and breast cancer risk in the South Asian population by analyzing six studies with a total of 950 cases and 882 controls. The polymorphism showed significant risk association in terms of three genetic models (allele model, dominant model and co-dominant model 2). The other three genetic models (co-dominant model 1, co-dominant model 3 and recessive model) also showed a higher risk, but the result was not statistically significant. According to the result, *TP53* codon 72 polymorphism is a substantial risk factor for the development of breast cancer in the South Asian population.

Similarly, *TP53* codon 72 polymorphism is susceptible to cause lung cancer, according to many previous studies (Hossain et al., 2017; Pierce et al., 2000; Hiraki et al., 2003; Sakiyama et al., 2005; Zhang et al., 2006; Jung et al., 2008; Piao et al., 2011). The polymorphism of *p53* codon 72 Arg/Pro also has been demonstrated to modify the risk for lung cancer among South Asians in many previous case-control studies. Several previous studies demonstrated that the* p53* codon 72 Arg/Pro polymorphism was not significantly associated with lung cancer susceptibility (Pierce et al., 2000; Piao et al., 2011). On the contrary, the *p53* codon 72 polymorphism was confirmed to be related to an elevated risk of lung cancer in other studies Chua et al., 2010; Hossain et al., 2017; Hiraki et al., 2003; Sakiyama et al., 2005; Zhang et al., 2006; Jung et al., 2008). Different conflicting results may be results from the insufficient sample size, variable genotyping method or different study design. To evaluate the actual association between *TP53* codon 72 polymorphism and lung cancer in the South Asian population, we performed a meta-analysis based on 6 articles with a total of 975 cases and 1397 controls. The polymorphism showed a significantly increased risk of lung cancer in various genetic models. Allele, co-dominant genetic models 1-3, dominant model and recessive showed a significantly increased risk for the development of lung cancer (p<0.05). The overdominant model showed no risk for the development of lung cancer in the South Asian population. 

Our study findings have some limitations, although we systemically searched for eligible publications. Firstly, only six studies of breast cancer and six studies of lung cancer were included as no other relevant studies were found. Secondly, only the South Asian cases and controls were included in the study. Therefore, our pooled results may not be extrapolated to other ethnic groups. Despite these limitations, this is the first meta-analysis of the South Asian population to evaluate the association of p53Arg72Pro polymorphism with breast and lung cancer. We performed the heterogeneity, sensitivity and publication bias analyses that are strengths of this meta-analysis.

In conclusion, this combined meta-analysis indicates that the TP53 codon 72 polymorphism increases the risk of both breast cancer and lung cancer in the South Asian population. 

## References

[B1] Akkiprik M, Sonmez O, Gulluoglu BM (2009). Analysis of p53 gene polymorphisms and protein over-expression in patients with breast cancer. Pathol OncolRes.

[B2] Aziz I, Rashid MU, Sultan F (2013). Frequency of pro allele on codon 72 of TP53 in female breast cancer patients of Pakistan: Molecular Stress or Geography. Pakistan J Zool.

[B3] Baynes C, Healey CS, Pooley KA (2007). Common variants in the ATM, BRCA1, BRCA2, CHEK2 and TP53 cancer susceptibility genes are unlikely to increase breast cancer risk. Breast Cancer Res.

[B4] Buyru N, Tigli H, Dalay N (2003). P53 codon 72 polymorphism in breast cancer. Oncol Rep.

[B5] Chowdhury MK, Moniruzzaman M, Emran AA (2015). TP53 codon 72 polymorphisms and lung cancer risk in the Bangladeshi population. Asian Pac J Cancer Prev.

[B6] Chua HW, Ng D, Choo S (2010). Effect of MDM2 SNP309 and p53 codon 72 polymorphisms on lung cancer risk and survival among non-smoking Chinese women in Singapore. BMC Cancer.

[B7] Damin AP, Frazzon AP, Damin DC (2006). Evidence for an association of TP53 codon 72 polymorphism with breast cancer risk. Cancer Detect Prev.

[B9] Goncalves ML, Borja SM, Cordeiro JA (2014). Association of the TP53 codon 72 polymorphism and breast cancer risk: a meta-analysis. Springerplus.

[B10] Hashemi M, Bahari G, Markowski J (2018). Association of PDCD6 polymorphisms with the risk of cancer: Evidence from a meta-analysis. Oncotarget.

[B11] Henriquez-Hernandez LA, Murias-Rosales A, Hernandez Gonzalez A (2009). Gene polymorphisms in TYMS, MTHFR, p53 and MDR1 as risk factors for breast cancer: A case-control study. Oncol Rep.

[B12] Hiraki A, Matsuo K, Hamajima N (2003). Different risk relations with smoking for non-small-cell lung cancer: Comparison of TP53 and TP73 Genotypes. Asian Pac J Cancer Prev.

[B13] Hossain A, Murshid GMM, Zilani MNH (2017). TP53 codon 72 polymorphism and breast cancer risk in Bangladeshi population. Breast Cancer.

[B14] Hou J, Jiang Y, Tang W, Jia S (2013). p53 codon 72 polymorphism and breast cancer risk: A meta-analysis. Exp Ther Med.

[B15] Huang XE, Hamajima N, Katsuda N (2003). Association of p53 codon Arg72Pro and p73 G4C 14-to-A4T14 at exon 2 genetic polymorphisms with the risk of Japanese breast cancer. Breast Cancer.

[B16] Ihsan R, Devi TR, Yadav DS (2011). Investigation on the role of p53 codon 72 polymorphism and interactions with tobacco, betel quid, and alcohol in susceptibility to cancers in a high-risk population from North East India. DNA Cell Biol.

[B17] Islam MS, Ahmed MU, Sayeed MS (2013). Lung cancer risk in relation to nicotinic acetylcholine receptor,CYP2A6 and CYP1A1 genotypes in the Bangladeshi population. Clin Chim Acta.

[B18] Jain N, Singh V, Hedau S (2005). Infection of human papillomavirus type 18 and p53 codon 72 polymorphism in lung cancer patients from India. Chest.

[B19] Jung HY, Whang YM, Sung JS (2008). Association study of TP53 polymorphisms with lung cancer in a Korean population. J Hum Genet.

[B20] Kalemi TG, Lambropoulos AF, Gueorguiev M (2005). The association of p53 mutations and p53 codon 72, Her 2 codon 655 and MTHFR C677T polymorphisms with breast cancer in Northern Greece. Cancer Lett.

[B21] Katiyar S, Thelma BK, Murthy NS (2003). Polymorphism of the p53 codon 72 arg/pro and the risk of HPV type 16/18-associated cervical and oral cancer in India. Mol Cell Biochem.

[B22] Khadang B, Fattahi MJ, Talei A, Dehaghani AS, Ghaderi A (2007). Polymorphism of TP53 codon 72 showed no association with breast cancer in Iranian women. Cancer Genet Cytogenet.

[B23] Khan MH, Khalil A, Rashid H (2015). Evaluation of the p53 Arg72Pro polymorphism and its association with cancer risk: a HuGE review and meta-analysis. Genet Res (Camb).

[B24] Li T, Lu ZM, Guo M (2002). p53 Codon 72 polymorphism (C/G) and the risk of human papillomavirus-associated carcinomas in China. Cancer.

[B25] Liu D, Wang F, Guo X (2013). Association between p53 codon 72 genetic polymorphisms and tobacco use and lung cancer risk in a Chinese population. Mol Biol Rep.

[B26] Mabrouk I, Baccouche S, El-Abed R (2013). No evidence of correlation between p53 codon 72 polymorphism and risk of bladder or breast carcinoma in Tunisian patients. Ann N Y Acad Sci.

[B27] Mantovani F, Collavin L, Del Sal G (2019). Mutant p53 as a guardian of the cancer cell. Cell Death Differ.

[B28] Meek DW (2015). Regulation of the p53 response and its relationship to cancer. Biochem J.

[B29] Mehra NK (2010). Defining genetic architecture of the populations in the Indian subcon-tinent: impact of human leukocyte antigen diversity studies. Indian J Hum Genet.

[B30] Mostaid MS, Ahmed MU, Islam MS, Bin Sayeed MS, Hasnat A (2014). Lung cancer risk in relation to TP53 codon 47 and codon 72 polymorphism in Bangladeshi population. Tumor Biol.

[B31] Muller PA, Vousden KH (2014). Mutant p53 in cancer: New functions and therapeutic opportunities. Cancer Cell.

[B32] Murata M, Tagawa M, Kimura M (1996). Analysis of a germ line polymorphism of the p53 gene in lung cancer patients; Discrete results with smoking history. Carcinogenesis.

[B33] Oren M, Rotter V (2010). Mutant p53 gain-of-function in cancer. Cold Spring Harb Perspect Biol.

[B34] Petitjean A, Achatz MI, Borresen-Dale AL, Hainaut P, Olivier M (2007). TP53 mutations in human cancers: Functional selection and impact on cancer prognosis and outcomes. Oncogene.

[B35] Piao JM, Kim HN, Song HR (2011). p53 codon 72 polymorphism and the risk of lung cancer in a Korean population. Lung Cancer.

[B36] Pierce LM, Sivaraman L, Chang W (2000). Relationships of TP53 codon 72 and HRAS1 polymorphisms with lung cancer risk in an ethnically diverse population. Cancer Epidemiol Biomarkers Prev.

[B37] Rivlin N, Brosh R, Oren M, Rotter V (2011). Mutations in the p53 tumor suppressor gene: Important milestones at the various steps of tumorigenesis. Genes Cancer.

[B38] Saikia BJ, Das M, Sharma SK (2014). Association of a p53 codon 72 gene polymorphism with environmental factors and risk of lung cancer: A case control study in Mizoram and Manipur, a high incidence region in North East India. Asian Pac J Cancer Prev.

[B39] Sakiyama T, Kohno T, Mimaki S (2005). Association of amino acid substitution polymorphisms in DNA repair genes TP53, POLI, REV1 and LIG4 with lung cancer risk. Int J Cancer.

[B40] Schmidt MK, Reincke S, Broeks A (2007). Do MDM2 SNP309 and TP53 R72P interact in breast cancer susceptibility? A large pooled series from the breast cancer association consortium. Cancer Res.

[B41] Shabnaz S, Ahmed MU, Islam MS (2016). Breast cancer risk in relation to TP53 codon 72 and CDH1 gene polymorphisms in the Bangladeshi women. Tumor Biol.

[B42] Sharma S, Sambyal V, Guleria K (2014). TP53 polymorphisms in sporadic North Indian breast cancer patients. Asian Pac J Cancer Prev.

[B43] Sjalander A, Birgander R, Hallmans G (1996). P53 Polymorphisms and haplotypes in breast cancer. Carcinogenesis.

[B44] Sreeja L, Syamala V, Raveendran PB (2008). p53 Arg72Pro polymorphism predicts survival outcome in lung cancer patients in Indian population. Cancer Invest.

[B45] Suresh K, Venkatesan R, Chandirasekar R, Kumar BL, Sasikala K (2011). Association of Trp53 arg72pro polymorphic variants with breast cancer - A case control study in South Indian population. Biol Med.

[B46] Syeed N, Sameer AS, Abdullaha S, Husain SA, Siddiqi MA (2010). A case-control study of TP53 R72P polymorphism in the breast cancer patients of ethnic Kashmiri population. World J Oncol.

[B47] Tilak AR, Kumar S, Pant MC, Mathur N, Kumar A (2013). Polymorphism Arg72Pro of p53 confers susceptibility to squamous cell carcinoma of lungs in a North Indian population. DNA Cell Biol.

[B48] Tommiska J, Eerola H, Heinonen M (2005). Breast cancer patients with p53 pro72 homozygous genotype have a poorer survival. Clin Cancer Res.

[B49] Wang YC, Chen CY, Chen SK, Chang YY, Lin P (1999). p53 Codon 72 polymorphism in Taiwanese lung cancer patients: Association with lung cancer susceptibility and prognosis. Clin Cancer Res.

[B50] Zhang X, Miao X, Guo Y (2006). Genetic polymorphisms in cell cycle regulatory genes MDM2 and TP53 are associated with susceptibility to lung cancer. Hum Mutat.

